# Iterative Pulse–Echo Tomography for Ultrasonic Image Correction

**DOI:** 10.3390/s24061895

**Published:** 2024-03-15

**Authors:** Yuchen Zengqiu, Wentao Wu, Ling Xiao, Erlei Zhou, Zheng Cao, Jiadong Hua, Yue Wang

**Affiliations:** 1 Institute of Acoustics, Chinese Academy of Sciences, Beijing 100190, China; zengqyc@mail.ioa.ac.cn (Y.Z.); xling@mail.ioa.ac.cn (L.X.); zhouerlei@mail.ioa.ac.cn (E.Z.); caozheng@mail.ioa.ac.cn (Z.C.); wangyue@mail.ioa.ac.cn (Y.W.); 2 University of Chinese Academy of Sciences, Beijing 100049, China; 3 School of Reliability and Systems Engineering, Beihang University, Beijing 100191, China; huajiadong@buaa.edu.cn

**Keywords:** ultrasound tomography, aberration correction, ultrasound imaging

## Abstract

Acoustic aberration, caused by the uneven distribution of tissue speed-of-sound (SoS), significantly reduces the quality of ultrasound imaging. An important approach to mitigate this issue is imaging correction based on local SoS estimation. Computed ultrasound tomography in echo mode (CUTE) is an SoS estimation method that utilizes phase-shift information from ultrasound pulse–echo signals, offering both practical utility and computational efficiency. However, the traditional single-pass CUTE often suffers from poor accuracy and robustness. In this paper, an advanced approach known as iterative CUTE is introduced, which refines SoS estimates through iterative correction of errors and noise, addressing the limitations of traditional single-pass methods. It is argued that traditional precision indicators like root mean square error (RMSE) fall short in adequately reflecting the quality of SoS estimates for imaging correction, and coherence factor (CF) is proposed as a more indicative metric. Performance validation of the iterative CUTE algorithm was conducted using a simulation and agar phantom experiment. The results indicated that the iterative CUTE approach surpasses the single-pass approach, enhancing the average CF for SoS estimates by up to 18.2%. In phantom experiments, imaging corrected with SoS estimates from iterative CUTE reduced the Array Performance Index (API) by up to 40% compared to traditional methods.

## 1. Introduction

Medical ultrasound imaging is pivotal in diagnostics, offering a non-invasive, real-time visualization of internal body structures, facilitating early detection and management of various health conditions. Its safety, accessibility, and cost-effectiveness make it an indispensable tool in modern healthcare. One of the most significant factors limiting the quality of ultrasound imaging is image degradation caused by acoustic aberration; this occurs during ultrasound wave propagation in human tissues with unknown speed-of-sound (SoS) distribution. In various human tissues, variation in SoS can reach up to several percentage points, causing distortion of the ultrasound wavefront as it is transmitted into and reflected back from the tissue by the transducer. This distortion is referred to as acoustic aberration. In the absence of prior information, ultrasound imaging is typically based on the assumption of a uniform SoS distribution in the tissue, e.g., 1540 m/s. The mismatch between this presumed SoS and the actual SoS distribution can lead to defocusing during the transmission (Tx) and reception (Rx) processes, thereby limiting the resolution and contrast of the images [1,2,3].

Numerous techniques and models have been proposed to mitigate the image degradation that is caused by acoustic aberration [4,5,6,7,8,9]. A typical example is the near-field phase screen model, which simplifies the effect of acoustic aberration caused by uneven SoS distribution as a phase screen located in front of the ultrasound transducer [6]. This method estimates the appropriate time delay correction based on the phase relations of echo signals. Building on this, a method that uses narrow-focused beams to generate echo was proposed to increase echo signal coherence [7]. The fundamental limitation for this kind of method is that, when imaging more complex human tissues, the spatial variation of required time delay corrections for imaging becomes challenging to describe solely using a simple phase screen model. In such cases, estimating the local SoS distribution within human tissue becomes crucial for ultrasound imaging correction.

Transmission-mode ultrasound tomography is considered to be one of the most widely used techniques for SoS estimation. This technique utilizes the time-of-flight and power attenuation information from ultrasound signals transmitted through the tissue [10,11,12]. The premise for transmission-mode SoS estimation is that such human tissues is fully permeable to ultrasonic waves. Additionally, the reception of ultrasound transmission waves typically requires special hardware such as a circular fixture and a water tank. These requirements make it challenging to extend transmission-mode ultrasound tomography from applications like breast imaging to SoS estimation in conventional ultrasound imaging.

Efforts have been made to make use of the information from pulse–echo signals obtained using handheld ultrasound probes to reconstruct the SoS distribution within the tissues, which greatly extends the applicability of SoS estimation [9,13,14,15]. More recently, it has been noticed that, when ultrasonic pulses are transmitted with different steering angles, the phase shift between echo signals corresponding to these transmissions could be used for tomographic reconstruction. Building upon this, a method called computed ultrasound tomography in echo mode (CUTE) was proposed [16], which utilizes pulse–echo ultrasound image from different transmissions to estimate local SoS information in the Fourier domain. Subsequently, an implementation of CUTE in the spatial domain was proposed, addressing the issue of artifacts in Fourier domain SoS estimation [17]. A series of improved algorithms was proposed [18,19,20,21], focusing on phase-shift extraction and different types of transmissions, further enhancing the SoS estimation performance of the CUTE algorithm. Moreover, Jaeger et al. [8] and Rau et al. [22] independently validated the feasibility of imaging correction based on SoS estimation using gel phantoms and organic tissues. The results indicated a significant improvement in the quality of ultrasound images compared to imaging strategies using a single presumed SoS.

The primary constraints in utilizing SoS estimation for imaging correction are the robustness and accuracy of the SoS estimates. These can be influenced by various factors in the implementation of the CUTE algorithm. For instance, different regularization strategies during inversion have a substantial impact on the quality of the SoS inversion result [19]. A study conducted by Beuret et al. [23] also indicated that the precision of local SoS estimation depends on the disparity between the presumed SoS for beam-forming and actual SoS; the greater the difference, the poorer the accuracy of the SoS estimate. Additionally, the efficacy of the CUTE algorithm in estimating SoS within human tissues is often suboptimal when faced with inclusions of irregular shapes [20].

To address the various errors present in the SoS estimation of the current CUTE algorithm, this paper proposes an iterative CUTE algorithm that incorporates increment SoS correction into the CUTE framework suggested by Rau et al. [20]. Through the iterative optimization of SoS estimate, this approach aims to circumvent and rectify issues in the CUTE algorithm, such as regularization, susceptibility to SoS deviation, and strong dependence on the shape of inclusions. Furthermore, the current evaluation of SoS estimation typically relies on precision metrics such as root mean squared error (RMSE). However, the RMSE of SoS estimate often fails to accurately reflect how imaging correction based on SoS estimate influences image quality. To address this limitation, the use of the coherence factor (CF) as a metric was introduced to assess the effectiveness of employing SoS estimate for ultrasound imaging correction. This metric is considered to be more rational compared to precision metrics such as RMSE, as it offers a more nuanced evaluation of the impact of imaging correction using SoS estimation on image quality.

## 2. Iterative CUTE Algorithm

In B-mode ultrasound imaging, a series of differently oriented ultrasound waves is first transmitted by a ultrasound transducer. The propagation time, also known as time of flight (ToF), between the transducer and each imaging point during transmission and reception is calculated based on the propagation path. The echo signals are then delayed using the calculated ToF and superimposed at each point in the region-of-interest (ROI) to form an ultrasound image.

When there is a disparity between the actual SoS distribution *c* in the tissue and the presumed SoS $c_{0}$ used during ultrasound imaging, the actual ToF of ultrasound waves in the tissue will deviate from the presumed ToF, which is used as time delay during beam-forming. This deviation is referred to as aberration delay τ. Assuming straight-line propagation paths for ultrasound waves from the transducer to the imaging point $\vec{r}$ and back to the transducer after reflection by a scatter located at the imaging point, the aberration delay magnitude for a pulse echo is determined after beam-forming the reflected signal. This magnitude is defined by the line integral of the difference between the true slowness (inverse of SoS) and the slowness estimates along the wave-propagation path,

(1) $$\begin{array}{cc} \tau (\vec{r}) & =\int_{l^{Tx}}\Delta \sigma dl+\int_{l^{Rx}}\Delta \sigma dl \end{array}$$

(2) $$\begin{array}{cc} \Delta \sigma & =\frac{1}{c}-\frac{1}{c_{0}} \end{array}$$

where $l^{Tx}$ and $l^{Rx}$ denotes the wave-propagation path from the transducer to $\vec{r}$ and from $\vec{r}$ back to the transducer during Tx and Rx, respectively. For a single ultrasound image beam-formed using echo signals of a single Tx, it is difficult to measure the absolute values of the aberration delay directly. However, for two ultrasound images beam-formed using the echo signals of different Txs, the aberration delay distribution of these images will differ due to the different wave-propagation paths. Furthermore, if the ultrasound image are beam-formed using complex (analytic) RF signals, this difference in aberration delay will manifest as phase shift between the two complex RF-mode (crf-mode) images, $I_{tx_{1}}\left(\vec{r}\right)$ and $I_{tx_{1}}\left(\vec{r}\right)$, which can be extracted using a point-wise Hermitian product [18].

(3) $$\Delta \tau \left(\vec{r},tx_{1},tx_{2}\right)=\tau \left(\vec{r},tx_{1}\right)-\tau \left(\vec{r},tx_{2}\right)=\arg\left(I\left(\vec{r},tx_{1}\right)\bar{I}\left(\vec{r},tx_{2}\right)\right)/\left(2\pi f\right)$$

Thus, a forward model that relates local SoS distribution and phase shift based on the aforementioned line integral relation is formulated:

(4) $$\begin{array}{cc} \Delta \tau (\vec{r},tx_{1},tx_{2})= & \tau \left(\vec{r},tx_{1}\right)-\tau \left(\vec{r},tx_{2}\right) \end{array}$$

(5) $$\begin{array}{cc} = & \int_{l_{tx_{1}}^{Tx}}\Delta \sigma dl+\int_{l_{tx_{1}}^{Rx}}\Delta \sigma dl-\left(\int_{l_{tx_{2}}^{Tx}}\Delta \sigma dl+\int_{l_{tx_{2}}^{Rx}}\Delta \sigma dl\right) \end{array}$$

### 2.1. Numerical Realization and Inversion of Forward Model

To facilitate the implementation of the forward model, as well as to further obtain estimates for tissue SoS by inversing the forward model, it is necessary to express the forward model in a discretized numerical form. The ROI is divided into a 2D Cartesian grid with dimensions of $N_{j}\times N_{k}$. The slowness deviation $\Delta \sigma (\vec{r})$ and phase shift $\Delta \tau (\vec{r},tx_{1},tx_{2})$ within the ROI are discretized into $\Delta \sigma_{j,k}$, $\Delta \tau_{j,k}\left(tx_{1},tx_{2}\right)$, indexed by the grid-point indices (j,k). Using bilinear interpolation, the numerical computation of the line integral of slowness deviation is achieved in a form of weighted summation.

(6) $$\int_{l}\Delta \sigma d\Leftrightarrow \sum_{j^{\prime },k^{\prime }}\Delta \sigma_{j^{\prime },k^{\prime }}w_{j^{\prime },k^{\prime }}^{l}$$

**Figure 1:**
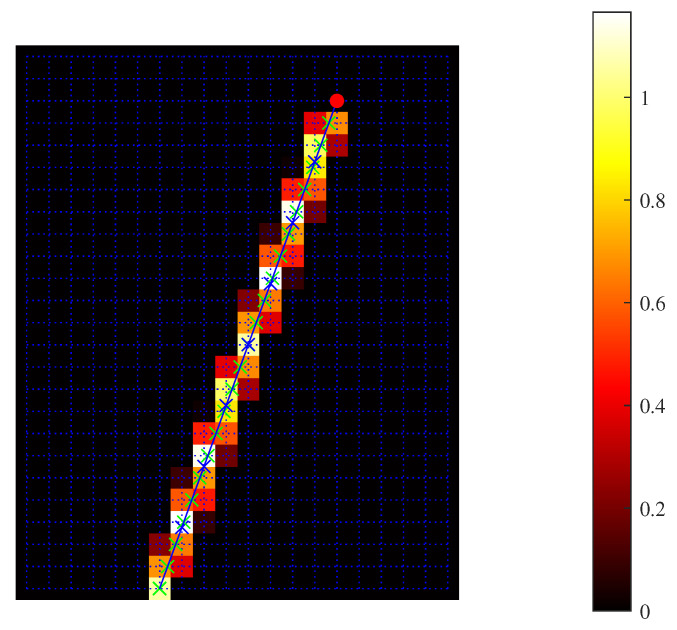
Illustration of the weights discretized from a propagation path calculated using bilinear interpolation.

The integral relationship between phase shift and slowness deviation expressed in Equation (4) is discretized as follows:

(7) $$\Delta \tau_{j,k}\left(tx_{1},tx_{2}\right)=\sum_{j^{\prime },k^{\prime }}\Delta \sigma_{j^{\prime },k^{\prime }}\left(w_{j^{\prime },k^{\prime }}^{l_{tx1}^{Tx}}\left(tx_{1},j,k\right)+w_{j^{\prime },k^{\prime }}^{l_{tx1}^{Rx}}\left(tx_{1},j,k\right)-w_{j^{\prime },k^{\prime }}^{l_{tx2}^{Tx}}\left(tx_{2},j,k\right)-w_{j^{\prime },k^{\prime }}^{l_{tx2}^{Rx}}\left(tx_{2},j,k\right)\right)$$

where $w_{j^{\prime },k^{\prime }}^{l_{tx1}^{Tx}}\left(tx_{1},j,k\right)$ and $w_{j^{\prime },k^{\prime }}^{l_{tx1}^{Rx}}\left(tx_{1},j,k\right)$ denote the discretized gird weights representing Tx path $l_{tx_{1}}^{Tx}$ for the transducer to image grid point (j,k) and Rx paths $l_{tx_{1}}^{Rx}$ from that image grid point back to the transducer set by transmission $tx_{1}$. The weights at each grid point are obtained through a bilinear interpolation [24]. Figure 1 shows an example of such line integral weights discretized from a propagation path.

**Figure 2:**
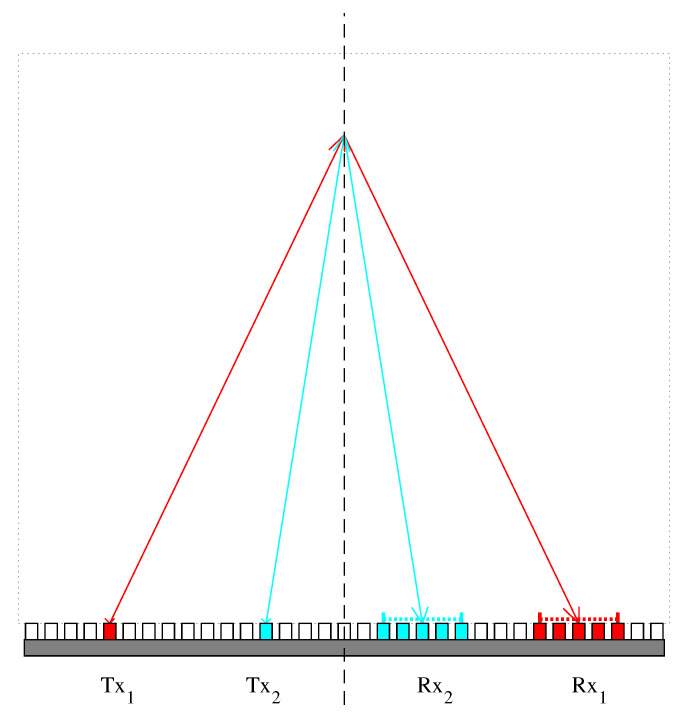
Apodization corresponding to CMA constraint. For two diverging wave transmissions, ${\mathrm{Tx}}_{1}$ and ${\mathrm{Tx}}_{2}$, to maintain a CMA of $\psi =0^{\circ }$, the corresponding reception apertures, ${\mathrm{Rx}}_{1}$ and ${\mathrm{Rx}}_{2}$, require the illustrated apodization.

For echo signals corresponding to different Txs, using a constant Rx aperture for beam-forming results in image rotation due to the varying directions of Tx propagation paths. This rotation reduces the coherence of echoes from different Txs after beam-forming, which negatively impacts phase-shift extraction. To mitigate the decorrelation effect and improve the quality of phase-shift extraction and the inversed SoS estimate, it is necessary to constrain the median steering angle between the Tx and Rx propagation path to remain constant. This constraint is referred to as the common middle angle (CMA) [25]. The ultrasound transmissions used in this paper are diverging waves (DWs) transmitted by different array elements. For this kind of transmission, the CMA constraint is achieved by a point-wise dynamic apodization, as illustrated in Figure 2.

After introducing the CMA constraint during beam-forming, correspondingly, the CMA term ψ is incorporated into the phase-shift extraction described in Equation (3) and discretized forward model described in Equation (6):

(8) $$\Delta \tau \left(\vec{r},tx_{1},tx_{2},\psi \right)=\tau \left(\vec{r},tx_{1},\psi \right)-\tau \left(\vec{r},tx_{2},\psi \right)=\arg\left(I\left(\vec{r},tx_{1},\psi \right)\bar{I}\left(\vec{r},tx_{2},\psi \right)\right)/\left(2\pi f\right)$$

(9) $$\begin{array}{cc} \Delta \tau_{j,k}\left(tx_{1},tx_{2},\psi \right)= & \sum_{j^{\prime },k^{\prime }}\left(\Delta \sigma_{j^{\prime },k^{\prime }}(w_{j^{\prime },k^{\prime }}^{l_{tx1}^{Tx}}\left(tx_{1},j,k\right)+w_{j^{\prime },k^{\prime }}^{l_{tx1}^{Rx}}\left(tx_{1},j,k,\psi \right)\right.\\ \; & \left.-w_{j^{\prime },k^{\prime }}^{l_{tx2}^{Tx}}\left(tx_{2},j,k\right)-w_{j^{\prime },k^{\prime }}^{l_{tx2}^{Rx}}\left(tx_{2},j,k,\psi \right)\right) \end{array}$$

where $w_{j^{\prime },k^{\prime }}^{l_{tx_{1}}^{Tx}}\left(tx1,j,k\right)$ denotes the weights corresponding to the transmission path $l_{tx_{1}}^{Tx}$ defined by transmission $tx_{1}$ and image point (j,k). $w_{j^{\prime },k^{\prime }}^{l_{tx_{1}}^{Rx}}\left(tx1,j,k,\psi \right)$ denotes the weights corresponding to the reception path $l_{tx_{1}}^{Rx}\left(\psi \right)$ defined by image point (j,k), transmission path $l_{tx_{1}}^{Tx}$, and CMA ψ.

Echo signals from a total of *n* DW transmissions $tx_{1},tx_{2},\ldots ,tx_{n}$ are acquired, and they are beam-formed point-by-point to produce crf-mode images under the constraint of *m* CMAs $\psi 1,\psi_{2},\ldots ,\psi_{m}$. For a crf-mode image sequence $I(tx_{1},\psi ),$$I\left(tx_{2},\psi \right),\ldots ,I\left(tx_{n},\psi \right)$ with the same CMA constraint ψ, the phase shift between each two consecutive images, $\Delta \tau_{j,k}\left(tx_{1},tx_{2},\psi \right),$$\Delta \tau_{j,k}\left(tx_{2},tx_{3},\psi \right),\ldots ,\Delta \tau_{j,k}\left(tx_{n-1},tx_{n},\psi \right)$, is extracted using a point-wise Hermitian product [18]. Vectorizing and combining these phase shifts, a column vector of phase shifts with a dimension of $m\left(n-1\right)N_{j}N_{k}\times 1$ is obtained. After writing Equation (9) in matrix notation, the matrix form of the discretized forward model linking slowness deviations and phase shift is formulated:

(10) $$\Delta \vec{\tau }=M\Delta \vec{\sigma }$$

where $\Delta \vec{\sigma }$ is the vectorized form of discretized slowness distribution $\Delta \sigma_{j,k}$. M is a matrix with dimensions $m\left(n-1\right)N_{j}N_{k}\times N_{j}N_{k}$, where each row vector of M, when subjected to an inner product with column vector $\Delta \vec{\sigma }$, corresponds to the discretized line integral process, as described in Equation (9). By inversing the phase-shift measurements using the forward model (Equation (10)), estimates of slowness deviation can be obtained. Subsequently, using the slowness deviation to correct the presumed SoS yields an estimation of SoS distribution within the tissue.

(11) $$\hat{c}=1/\left(\frac{1}{c_{0}}+\Delta \hat{\sigma }\right)$$

Due to the highly underdetermined nature of the forward model described above, to ensure the robustness of SoS estimation, the inverse of the forward model is performed using Tikhonov regularized pseudo-inverse [26], given by:

(12) $$\begin{array}{cc} \; & \Delta \vec{\hat{\sigma }}=M^{\mathrm{inv}}\Delta \vec{\tau }\\ \; & M^{\mathrm{inv}}={\left(M^{T}M+\gamma_{x}D_{x}^{T}D_{x}+\gamma_{z}D_{z}^{T}D_{z}\right)}^{-1}M^{T} \end{array}$$

which minimizes the expression

(13) $$C\left(\Delta \vec{\hat{\sigma }}\right)={∥\Delta \vec{\tau }-M\Delta \vec{\hat{\sigma }}∥}_{2}^{2}+\gamma_{x}{∥D_{x}\Delta \vec{\hat{\sigma }}∥}_{2}^{2}+\gamma_{z}{∥D_{z}\Delta \vec{\hat{\sigma }}∥}_{2}^{2}$$

where $D_{x}$ and $D_{z}$ are the difference operators in the x and z directions, respectively, used to ensure the smoothness of the inversed SoS estimate. $\gamma_{x}$ and $\gamma_{z}$ are the regularization parameters controlling the regularization strength on the slowness deviation in the x and z directions during the inversion process. Unlike traditional full-wave inversion tomographic algorithms that require specialized hardware to acquire signals of echoes from tissues in all directions, the algorithm described above estimates the local tissue SoS through the inversion of phase shift, relying solely on the use of pulse echo signals generated by a conventional handheld ultrasound transducer.

### 2.2. Iterative Correction of SoS Estimate

The accuracy of the SoS estimate obtained through the current CUTE algorithm is influenced by various factors. For instance, in the phase-shift extraction stage of CUTE algorithm, the numerical values of the extracted phase shift often deviate from the theoretical values predicted by the forward model, which worsens with greater deviation between presumed and true SoS [18]. Research by Rau et al. [27] has shown that different phase-shift extraction methods yield numerically inconsistent phase-shift measurement values. When inversed, this inconsistent phase shift will lead to a numerically inconsistent SoS estimate. Additionally, the regularized inverse strategy for phase shift also impacts SoS estimation. Weaker regularization can cause severe interference in slowness deviation estimate, leading to the distortion of the SoS estimate [16]. On the other hand, stronger regularization may result in underestimated slowness deviation, suppressing the corrections for the misestimated SoS.

A significant reason for the aforementioned series of errors in SoS estimation is the pursuit, within the current CUTE algorithm, of completing SoS estimation through a single correction process. The errors arising from various factors during single-pass SoS estimation can be better suppressed through multiple iterations of the SoS estimation results. Building upon this concept, an iterative CUTE algorithm is introduced that replaces the single-pass SoS correction with an iterative approach. Through repeated iterations and corrections, the SoS estimates are ultimately refined to more closely approximate the true values.

In the iterative CUTE algorithm, the time delay used for the echo signal beam-forming and phase-shift extraction is no longer calculated using a single presumed SoS through the geometric propagation path. Instead, it is computed using the current estimate of the local SoS distribution ${\hat{c}}_{n-1}$. The phase shift extracted in this manner reflects the difference between the current SoS estimate and the true SoS within the ROI $\Delta \sigma_{n}=\frac{1}{c_{\mathrm{real}}}-\frac{1}{{\hat{c}}_{n-1}}$. Consequently, the slowness deviation estimated through the inversion of this phase shift corrects the error between the current SoS estimate and the true SoS distribution. The use of this slowness deviation estimate to adjust the current SoS estimate results in an iteratively updated SoS estimate.

(14) $${\hat{c}}_{n}=1/\left(\frac{1}{{\hat{c}}_{n-1}}+\Delta {\hat{\sigma }}_{n}\right)$$

In each iteration of the process, the time delay for beam-forming, determined using the previous iteration’s SoS estimate, is obtained through the eikonal equation. For a medium with an SoS distribution c(x,z), the ToF from an array element positioned at $\left(x_{i},0\right)$, to each position (x,z) within the medium can be solved using an eikonal Equation, given the boundary condition $t(x_{i},0)=0$

(15) $$\sqrt{{\left(\frac{\partial t}{\partial x}\right)}^{2}+{\left(\frac{\partial t}{\partial z}\right)}^{2}}=\frac{1}{c(x,z)}$$

Repeating this process for each array element yields the ToF $t_{i}\left(x,z\right)$ for each element, which is used for beam-forming and phase-shift extraction. The eikonal equation is solved employing a multi-stencil fast marching method [28], allowing the efficient computation—through forward propagation—of ToF from a designated grid point to all other points within a discretized grid for a specified speed distribution. The iterative CUTE algorithm follows the process outlined below (Algorithm 1):
**Algorithm 1:** Iterative CUTE algorithm.
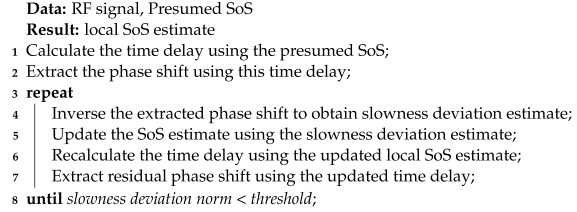


The iterative CUTE algorithm enables one to employ strong regularization constraints to suppress interference while iterative corrections progressively refine the SoS estimation results, narrowing the discrepancy between the estimated SoS distribution and the actual SoS. The iterative process continues until the norm of the slowness deviation resulting from phase-shift inversion is less than a specified threshold.

## 3. Simulation and Discussion

To validate the performance of the iterative CUTE algorithm in SoS estimation and its advantages compared to single-pass SoS estimation, simulations were conducted using a simulated linear ultrasound array. The iterative CUTE algorithm was applied to estimate SoS in phantoms containing inclusions of different shapes. A 128-element linear ultrasound array with a pitch of 300 μm was simulated using k-Wave [29]. The ultrasound excitation is a four-cycle Gaussian enveloped pulse with a central frequency of 5 MHz. The spatial sampling interval in the simulation was set to 75 μm, and the time sampling interval was 6.25 ns, corresponding to a sampling frequency of 160 MHz. Diverging waves were transmitted sequentially using elements 32–96, and the acquired array echo signals were beam-formed to produce crf-mode images with CMA constraints of −15°, 0°, and 15°. These parameters where chosen to ensure sufficient phase-shift information was obtained while minimizing the computational load. The beam-forming process was implemented through delay and sum, as follows:

(16) $$I\left(x,z\right)=\sum_{i}\alpha_{i}{\tilde{s}}_{i}\left({\hat{t}}_{0}\left(x,z\right)\right)$$

**Figure 3:**
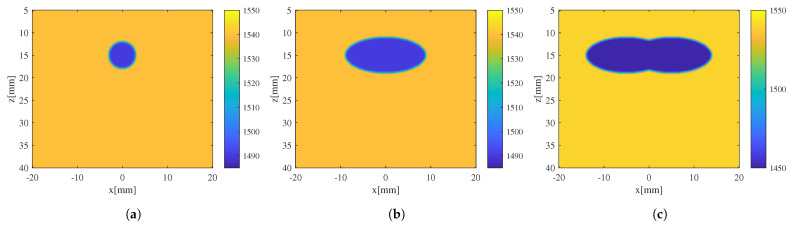
Simulated phantoms with inclusions of different shapes and SoS values: (**a**) phantom with cylindrical inclusion; (**b**) phantom with oval inclusion; (**c**) phantom with irregular strip-like inclusion.

The apodization weights $\alpha_{el}$ not only ensure adherence to the CMA constraints but also maintain a dynamic aperture apodization of F-number = 2. This serves to suppress side lobes and enhance image contrast. ${\tilde{s}}_{i}\left(t\right)$ represents the Hilbert transform of the echo signal received by the i-th array element, namely the complex RF signal. ${\hat{t}}_{0}\left(x,z\right)$ is the time delay calculated using the current SoS estimate. Phase-shift extraction was performed on every two consecutive crf-mode ultrasound images with the same CMA constraint using the point-wise Hermitian production described in Equation (3). For a sequence of Txs, the phase shift between the first and last Tx can be obtained by superposing the phase shift between every two consecutive Txs, $\begin{array}{cc} \Delta \tau_{1,N} & =\tau_{1}-\tau_{N}=\tau_{1}-\tau_{2}+\tau_{2}+\cdots +\tau_{N-1}-\tau_{N}\\ & =\Delta \tau_{1,2}+\cdots +\Delta \tau_{N-1,N} \end{array}$. By employing this superposition approach, it is possible to reduce the computation load of the inversion process without the loss of phase-shift information occurring, while improving the signal-to-noise ratio in phase-shift extraction. The extracted phase-shift distribution was inversed according to Equation (12) to obtain the slowness deviation estimate, which was used to correct the current SoS estimate, yielding the refined SoS estimate. Phantoms containing inclusions of cylindrical, oval, and irregular strip-like shapes were used for iterative CUTE SoS estimation. The different shapes, as shown in Figure 3, were chosen to verify the robustness of SoS estimation. Pulse–echo signals were generated using phantoms of these three different shapes, which were used to obtain initial estimates of SoS distribution by means of the phase-shift extraction and inversion described above. Subsequently, the initial SoS estimation results undergo three iterations of correction. On top of iterative CUTE performed using $c_{0}=1540\;m/s$, SoS estimations were performed on phantom with a cylindrical inclusion using the assumption of $c_{0}=1520\;m/s$ and $c_{0}=1560\;m/s$ to verify the algorithm’s robustness to SoS assumption.

### 3.1. Evaluation of the Effectiveness of Local SoS Correction

In the current research on ultrasound SoS estimation, the quality of SoS estimates is typically assessed using accuracy metrics such as signal-to-noise ratio (SNR) and root mean square error (RMSE). However, when applying SoS estimates for imaging correction—although, ideally, one would use SoS estimates with the highest precision to yield the optimal image—these metrics often struggle to reflect the actual impact of imperfect SoS estimates in real-world scenarios.

**Figure 4:**
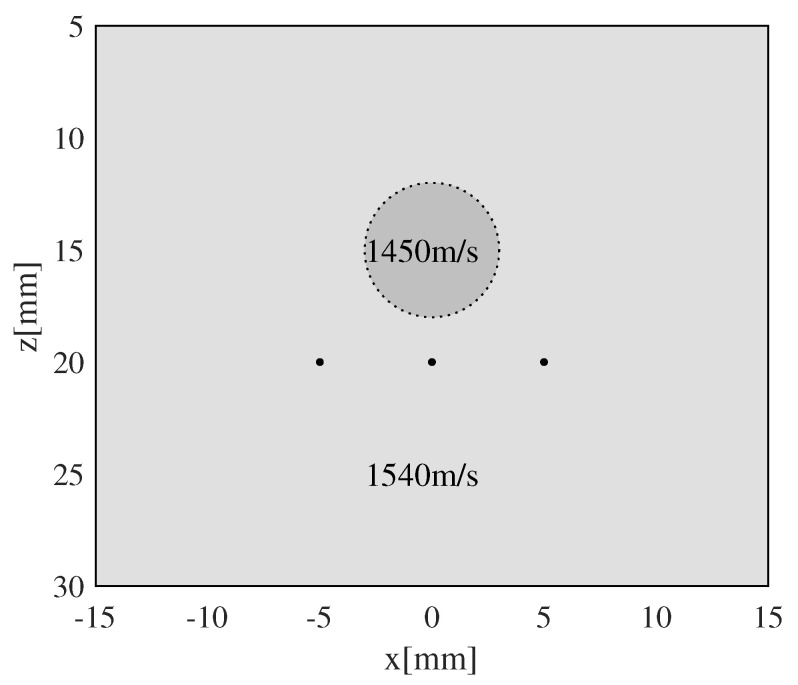
Sketch of phantom with a low-SoS cylindrical inclusion and three point targets.

A phantom with background SoS of 1540 m/s was simulated to demonstrate the limitations of accuracy metrics. A cylindrical inclusion with SoS of 1450 m/s was placed at a depth of 15 mm, along with three point targets placed at depths of 25 mm with lateral intervals of 5 mm. Figure 4 shows the sketch of this phantom.

**Figure 5:**
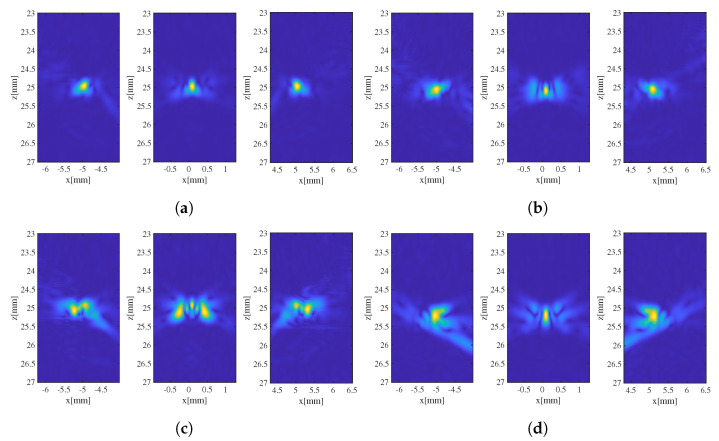
Image of 3 point targets. (**a**) Reference image beam-formed using real SoS. (**b**–**d**) Images beam-formed using SoS estimates 1, 2, and 3 (shown in Table 1), respectively.

Array echo signals corresponding to the phantom were simulated and acquired; these were beam-formed using three differently erroneous SoS estimates, as shown in Table 1, to form ultrasound images of these three point targets. Although the local errors between the three SoS estimates and the true SoS are not identical, the RMSE between the three SoS estimates and the true SoS are consistent at 9.7 m/s. The ultrasound images of the three point targets obtained using different SoS estimates are shown in Figure 5. It can be observed that, despite the same RMSE values corresponding to the three SoS estimates, the degradation of the images varies significantly due to the different distributions of specific local estimation errors. For the process of beam-forming correction using SoS estimates, an evaluation metric that directly reflects how the SoS estimates affect imaging was required.

The coherence factor, proposed by Mallart and Fink [30,31], is a metric widely used in ultrasound imaging to evaluate the quality of images obtained through beam-forming. It is defined as the ratio of the coherent summation intensity of the received array signals to the incoherent summation intensity.

(17) $$\mathrm{CF}=\frac{{\left(\sum_{i=1}^{N}s^{\left(i\right)}\left(t-\tau^{\left(i\right)}\right)\right)}^{2}}{N\sum_{i=1}^{N}s^{\left(i\right)}{\left(t-\tau^{\left(i\right)}\right)}^{2}}$$

The coherence factor, with values ranging between 0 and 1, indicates the quality of focus at a specific location—the higher the coherence factor of echo signals from a given position, the better the focus quality at that location. Due to the highly fluctuating nature of coherence factor obtained from real scatterer echo signals, this study utilizes a narrow-band analytical signal, $s\left(t\right)=G\left(t\right)\exp\left(2\pi f_{0}t\right)$, with the same frequency $f_{0}$ as the transmitted signal to simulate array echo signals. The prediction of the coherence factor is then calculated as an indicator to evaluate the effectiveness of local SoS correction in imaging. In the context of beam-forming—considering the significantly lower fluctuation rate of the complex envelope term G(t) compared to the carrier frequency term—the complex envelope term can be further simplified to a constant *G*. For a given position, (x,z), within the ROI, the true ultrasound propagation time, $t_{tof}^{\left(i\right)}$, between that location, the array element, *i*, and the time delay, $t_{bf}^{\left(i\right)}$—which is calculated based on the SoS estimate and used for beam-forming—is determined according to the eikonal equation. Thus, the expression for the echo signal generated at position (x,z) received by array element *i* after beam-forming can be simulated as

(18) $$s_{x,z}^{\left(i\right)}\left(t\right)=\alpha_{i}G\exp\left(2\pi if_{0}\left(t_{tof}^{\left(i\right)}\left(x,z\right)-t_{bf}^{\left(i\right)}\left(x,z\right)\right)\right)$$

where $\alpha_{i}$ denotes the apodization coefficient for the *i*-th element. In this paper, a dynamic aperture apodization with F-number = 2 is applied to each position in the imaging area. Substituting the echo signal expression into the coherence factor formula yields the predicted coherence factor based on the narrow-band analytic signal model:

(19) $$\begin{array}{cc} \; & \mathrm{CF}(x,z)= \end{array}$$

(20) $$\begin{array}{cc} & \frac{\left(\sum_{i=1}^{N}\alpha_{i}\exp\left(i2\pi f_{0}\left(t_{tof}^{\left(i\right)}\left(x,z\right)-t_{bf}^{\left(i\right)}\left(x,z\right)\right)\right)\right)\left(\sum_{i=1}^{N}\alpha_{i}\exp\left(-i2\pi f_{0}\left(t_{tof}^{\left(i\right)}\left(x,z\right)-t_{bf}^{\left(i\right)}\left(x,z\right)\right)\right)\right)}{{\left(\sum_{i=1}^{N}\alpha_{i}\right)}^{2}} \end{array}$$

### 3.2. Results and Discussion

**Figure 6:**
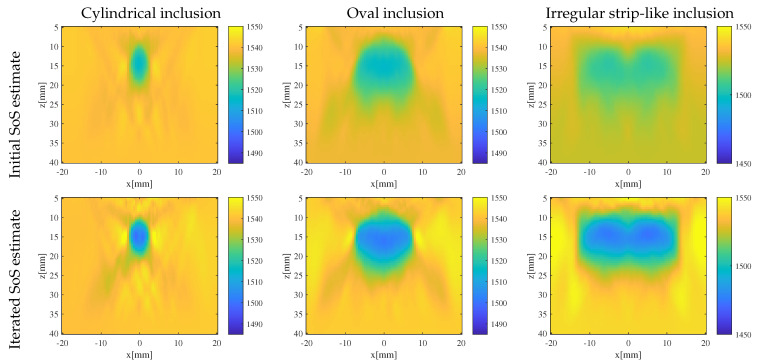
Initial and iterated SoS estimates for phantoms with inclusion of different shapes using non-iterative CUTE and the iterative CUTE algorithm.

The initial SoS estimates and iterated estimates for phantoms containing inclusions of the aforementioned three different shapes using traditional CUTE algorithm and the iterative CUTE algorithm are shown in Figure 6. For the phantom with a relatively simple cylindrical-shaped inclusion, the initial SoS estimate already closely approximates the true SoS distribution in terms of shape. However, there exists a significant difference in the value of inclusion SoS, deviating from the true value. As the iteration progresses, the inclusion SoS estimate gradually converges towards the true SoS.

In the case of the phantom with oval and irregular strip-like inclusions, the initial SoS estimate exhibit substantial differences in both numerical values and shapes compared to the true SoS distribution. However, with the ongoing iteration process, the SoS estimate gradually approaches the true values in terms of both shape and numerical values. In the meantime, the iterative process effectively corrects the misestimation of the phantom SoS behind larger-sized inclusions.

**Figure 7:**
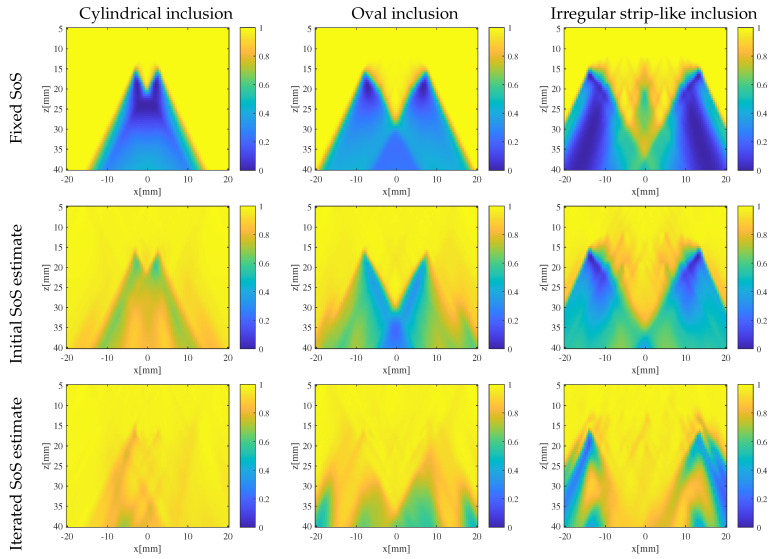
Coherence factor distribution prediction for different SoS estimates of phantoms with different inclusions.

To quantitatively investigate how the iterative process affects the quality of SoS estimates—especially in terms of improving image quality when applying SoS estimates to imaging correction—the CF distribution in the ROI for the three phantoms was calculated using a fixed SoS of 1540 m/s, the initial SoS estimates, and the iterated SoS estimates, as shown in Figure 7. In comparison to beam-forming using a fixed SoS, the initial SoS estimates already show noticeable improvement of CF during beam-forming. However, significant distortions are still noticeable in certain areas within the ROI.

As the iteration process proceeds, the quality of SoS estimation for each phantom significantly improves.

**Figure 8:**
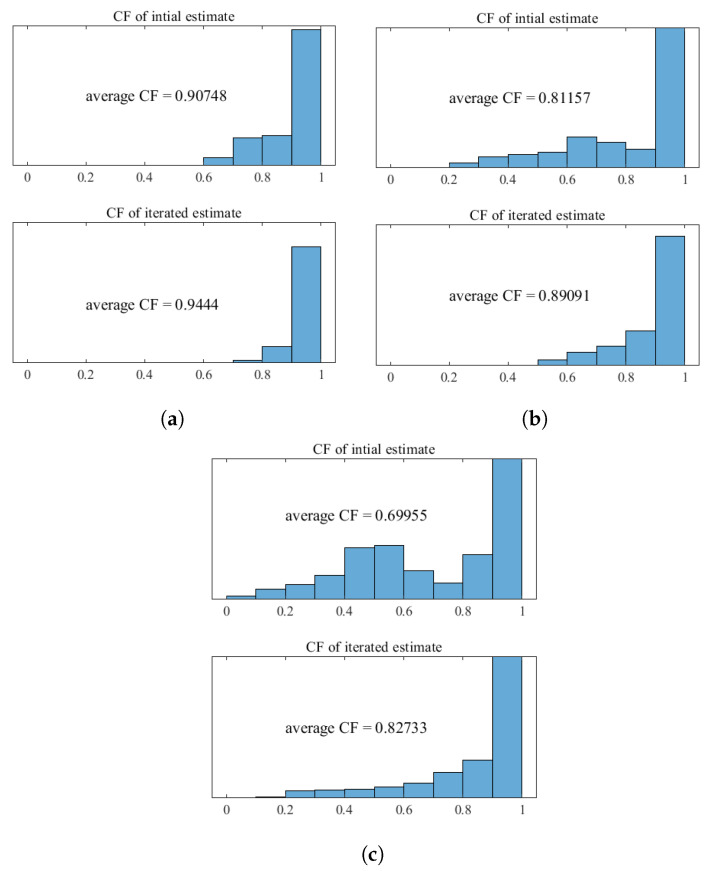
CF distribution of SoS estimate for each phantom before and after iteration: (**a**) phantom with cylindrical inclusion; (**b**) phantom with oval inclusion; (**c**) phantom with irregular strip-like inclusion.

For a detailed quantitative analysis of the coherence factor within the ROI, average coherence factor and CF distribution were calculated for the SoS estimates corresponding to each phantom. The CF average and distribution are displayed in Figure 8. The iteration process increases the average CF of three SoS estimates by 4%, 9.7%, and 18.2%, respectively. It can be observed that, as the iteration process progresses, with the increase in the average CF, a pronounced suppression of regions with low CF can be observed, especially in the case of the phantoms oval and irregular strip-like inclusions. After three iterations, the areas with severe image distortion, characterized by a CF value less than 0.5, have been largely eliminated. It can be noted that areas with the least CF improvement lie in the edges of the ROI. This is due to reduced phase-shift information produced by SoS misestimates on the edges of the ROI, which lead to insufficient SoS correction.

**Figure 9:**
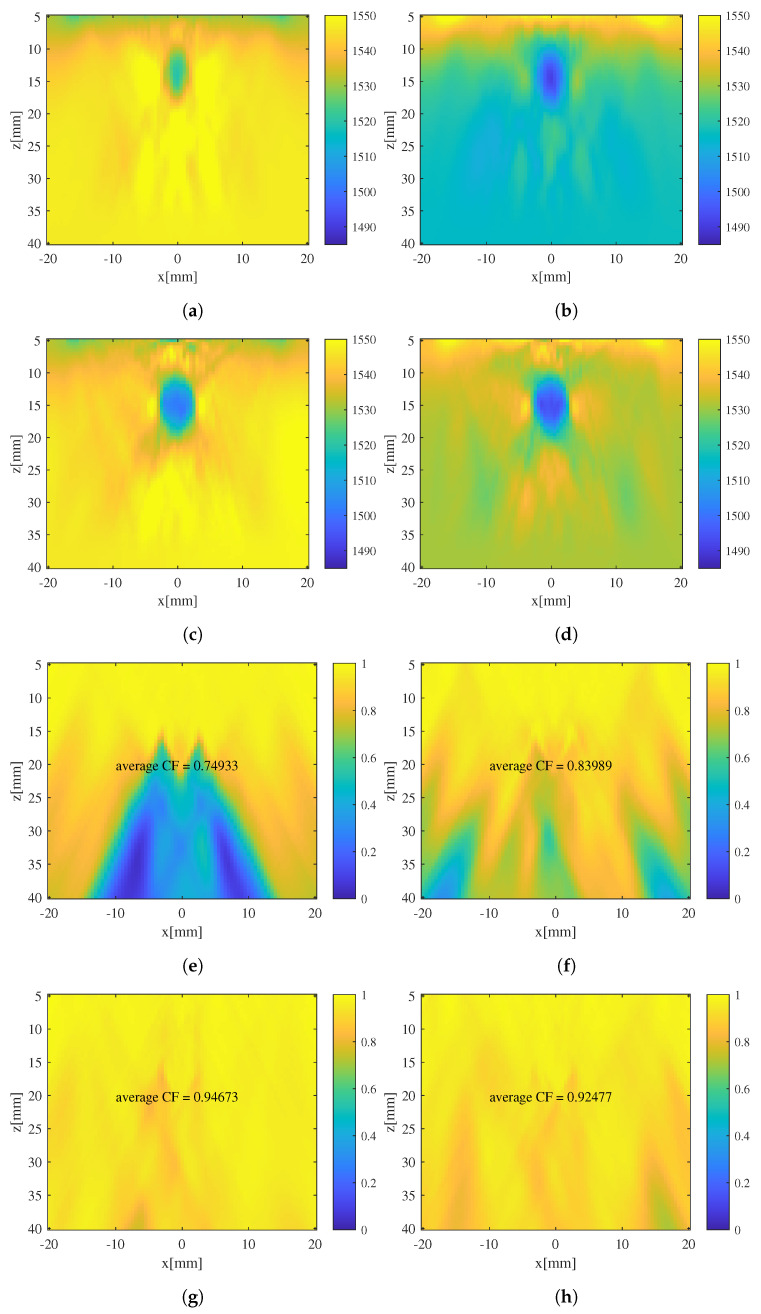
SoS estimates and CF distribution for phantom with cylindrical inclusion using different $c_{0}$. (**a**) Initial estimate using $c_{0}=1520\;m/s$. (**b**) Initial estimate using $c_{0}=1560\;m/s$. (**c**) Iterated estimate using $c_{0}=1520\;m/s$. (**d**) Iterated estimate using $c_{0}=1560\;m/s$. (**e**–**h**) CF distributions of (**a**–**d**).

Initial and iterated estimates of SoS distribution for phantoms with cylindrical inclusions obtained with different presumed SoS $c_{0}$ are displayed in Figure 9. It can be observed that, when there is a discrepancy between the presumed SoS values, $c_{0}$, a significant artifact can be observed in the initial SoS estimates. The average CF values for such SoS estimates are even worse those of a fixed SoS distribution $c_{0}=1540\;m/s$. As the iteration progresses, the SoS estimates converge toward the true value and the artifact is suppressed. Although the average numerical value of SoS estimates is slightly different from that obtained with $c_{0}=1540\;m/s$, the average CF is improved to the same level after SoS iteration. For the application of image correction, iterative CUTE algorithms demonstrates robustness with respect to SoS presumption $c_{0}$.

**Figure 10:**
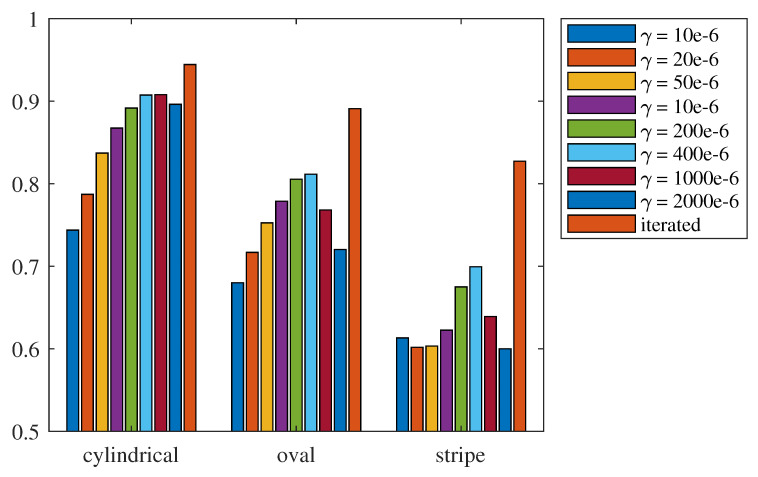
Average CF of SoS estimated using different regularization.

To verify whether the iterative CUTE algorithm can address the contradiction between the interference in SoS estimation and the insufficient SoS correction present in the non-iterative CUTE algorithm, phase shift extracted from echoes of the three phantoms were inversed using different regularization strengths to obtain single-pass SoS estimate using the non-iterative CUTE algorithm. The average CF of these SoS estimates were calculated and compared with the CF corresponding to the iterated SoS estimates produced by the iterative CUTE algorithm, as shown in Figure 10. For simplicity sake, the regularization in x and z direction are set to the same as $\gamma =\gamma_{x}=\gamma_{z}$.

It can be observed that, in the single-pass SoS estimation results, with lower regularization strength, the SoS estimate based on the phase-shift inversion exhibits strong interference, resulting in a relatively low average CF. As the regularization strength increases, the interference in SoS estimate is suppressed, leading to a rise in the average CF. However, with further increase in regularization strength, due to insufficient correction for areas with misestimated SoS, the average CF decreases again. Additionally, within the CUTE algorithm framework, the iterative approach to SoS estimation consistently outperforms the single-pass method in terms of quality, irrespective of the phantom inclusion’s shape. This is attributed to the iterative approach, which is able to effectively balance the interference suppression in SoS estimation of strong regularization and the sufficient SoS misestimation control of weak regularization.

Compared to single-pass SoS estimation, the iterative correction introduced in the CUTE algorithm offers the following advantages:

1. Targeted error reduction in SoS estimation: the iterative approach of the CUTE algorithm methodically narrows the discrepancy between actual and estimated SoS, minimizing the specific errors caused by these discrepancies and thereby enhancing the precision of the SoS estimation in a targeted manner.
2. Improved handling of regularization challenges: The iterative framework of the CUTE algorithm allows for iterative SoS correction with increased regularization strength. This approach mitigates interference related to insufficient regularization, as well as the under-correction of SoS results related to strong regularization.
3. Enhanced reconstruction of irregularly shaped inclusions: through iterative error correction, the iterative CUTE algorithm demonstrates more precise reconstructions that are closer to the true characteristics of these inclusions.

## 4. Validation Experiment

**Figure 11:**
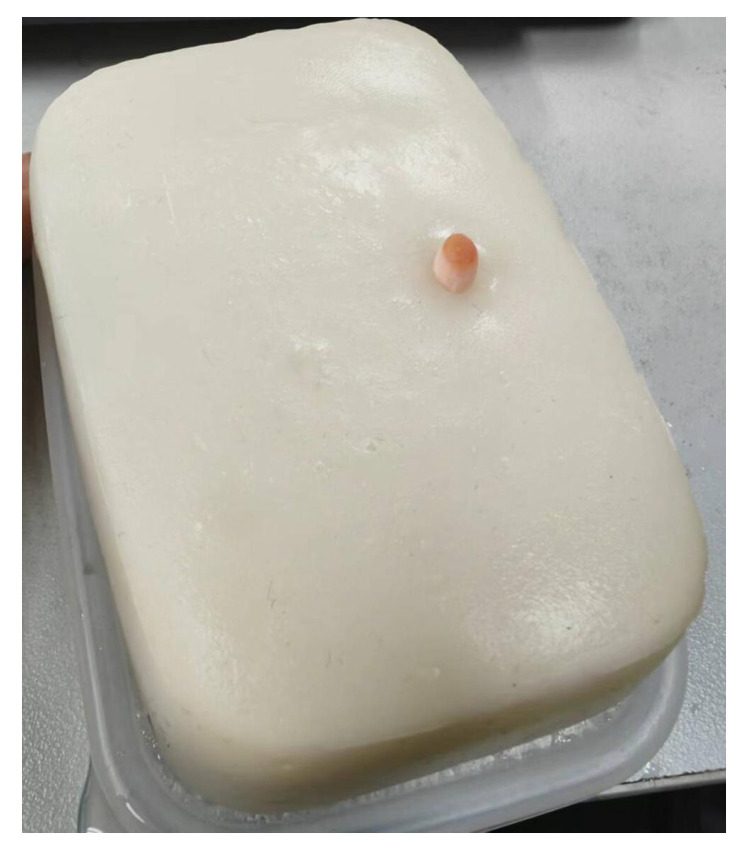
The agar phantom with a shredded ham strip as the high-speed inclusion.

To verify the effectiveness of the CUTE algorithm in real-world experiments, an agar phantom incorporating a shredded ham strip as the high-speed inclusion was created. Agar was melted by boiling in water at a ratio of 1:50, cooled to 60–70 °C, and mixed with a small amount of starch suspension (1:1) to increase the scattering coefficient of the agar material. This mixture was poured into a rectangular block mold. High-speed inclusions were created using ham with a higher SoS than agar. In ultrasound imaging, the agar block with added starch achieved a scattering intensity similar to the ham while maintaining good sound penetration. The agar phantom is shown in Figure 11. The SoS of the agar was approximately 1460 m/s, and the high-speed inclusion made of the ham material had an SoS of approximately 1500 m/s.

A 5L128 (128 elements) linear array probe was used for local SoS estimation and imaging of the phantom. The array element spacing was 0.312 mm, the element height was 10 mm, and the probe had a bandwidth of 3.5 MHz–6.5 MHz, with a center frequency of 5 MHz. The probe was positioned at the upper edge of the phantom, ensuring that the probe’s imaging plane of the ultrasound probe was perpendicular to the cylindrical inclusion. Echo signal acquisition was performed using a 32/128 channel ultrasound phased-array hardware system and data-acquisition software developed by the research group, with a sampling rate of 50 MSPS. Elements 32–96 on the linear array were sequentially excited to obtain full-aperture echo signals corresponding to the diverging waves emitted by each element.

**Figure 12:**
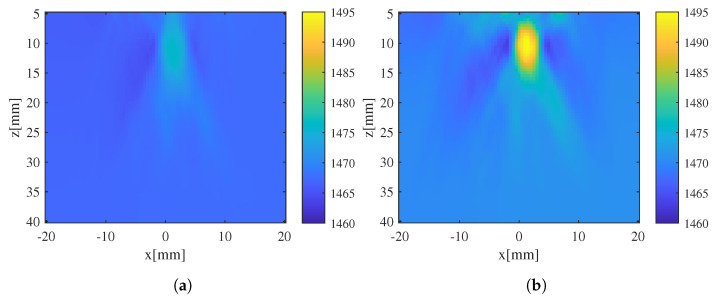
SoS estimate using CUTE and the results after iteration: (**a**) initial SoS estimate; (**b**) iterated SoS estimate.

For the collected array echo signals, the phase-shift distribution corresponding to the diverging waves between elements 32 and 96 $\Delta \tau_{32,36},\Delta \tau_{36,40},\ldots \Delta \tau_{92,96}$, with an interval of 4 elements, was initially extracted using a fixed SoS assumption. The initial SoS estimate was then obtained by inversing the phase-shift distribution. Subsequently, the SoS estimate was refined through 3 iterations of the previously described iterative correction process. The initial and iterated SoS estimates are shown in Figure 12. It can be observed that the initial SoS estimate, while indicating the location of the inclusion, still exhibited significant errors in estimating the shape and SoS value of the inclusion. Through the iteration and refinement of the SoS estimate, both the shape and the SoS value of the inclusion were effectively corrected. After 3 iterations, the SoS estimate of the agar phantom stabilized. Because the true agar SoS is unknown, some fluctuations of the background SoS can be noticed, similar to those shown in Figure 9.

**Figure 13:**
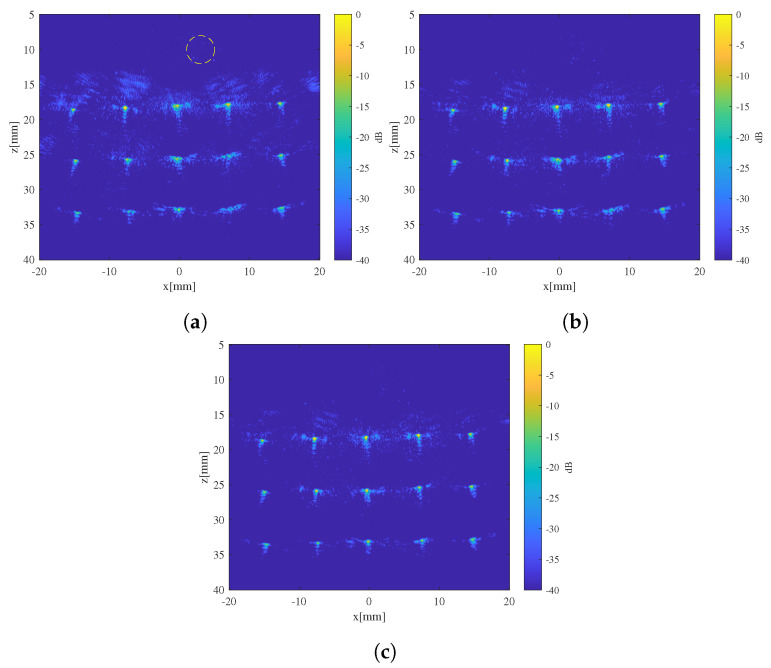
Ultrasound images beam-formed using different SoS estimates: (**a**) image beam-formed with a fixed SoS (1500 m/s); (**b**) image beam-formed with initial SoS estimate; (**c**) image beam-formed with iterated SoS estimate. The position of the inclusion is marked with a dashed line in (**a**).

To assess the impact of the SoS estimate on image quality when used for imaging correction, a point-scatter grid was created beneath the inclusion using equally spaced pins. The pins were arranged in a grid pattern of 3 rows and 5 columns, totaling 15 pins, with a spacing of 7.5 mm between rows and columns. After collecting full-matrix echo signals with the ultrasound probe, beam-forming was performed on the echo signals using the background SoS of the phantom, the initial SoS estimate, and the iterated SoS estimate (Figure 13). Using time delay calculated based on the method described in Section 2.2, beam-forming was performed to produce ultrasound images corresponding to different SoS estimates. All ultrasound images were normalized and logarithmically compressed with dynamic range of 40 dB.

For images beam-formed using a single, fixed SoS, significant defocusing can be observed for certain point targets. According to predictions previously made using coherence factor, for tissue with a cylindrical inclusion, the region directly beneath and diagonally beneath the inclusion exhibits the lowest coherence factors, indicating severe defocusing. In contrast, regions farther from the inclusion have higher coherence factors and the corresponding defocusing is negligible, which aligns with the actual imaging results.

**Figure 14:**
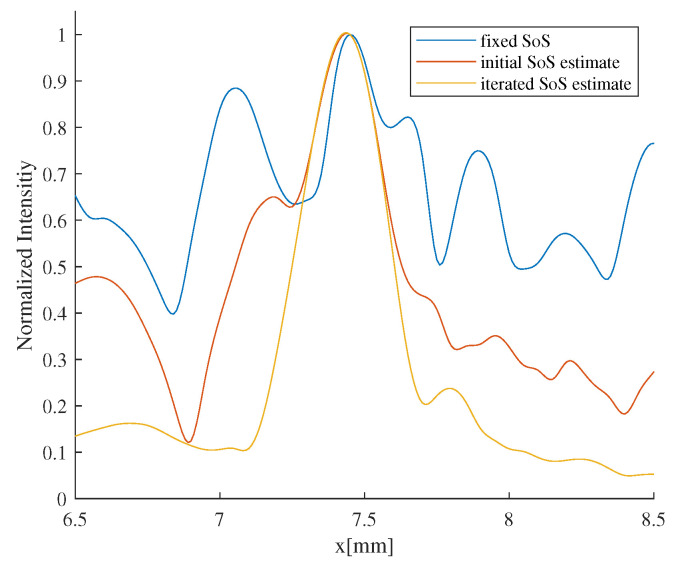
Normalized lateral image intensity distribution of aberrated point target.

After introducing the initial SoS estimates for imaging correction, some mitigation of defocusing was achieved. However, due to the still substantial differences between the SoS estimate and the true value, the imaging quality remained less than ideal. After 3 iterations, the SoS estimates converged towards the true SoS distribution, and the defocusing in the images beam-formed using iterated SoS estimate was essentially eliminated. Figure 14 illustrates the normalized lateral distribution of image intensity of a point target located at (7.5 mm, 33 mm), which suffered the most severe degradation, using different SoS estimates. Introducing the initial SoS estimate for imaging correction resulted in a 33% reduction in the full-width-at-half-maximum (FWHM) of target lateral intensity. A further decrease of 38% in FWHM was achieved with the introduction of iterated SoS estimates.

**Figure 15:**
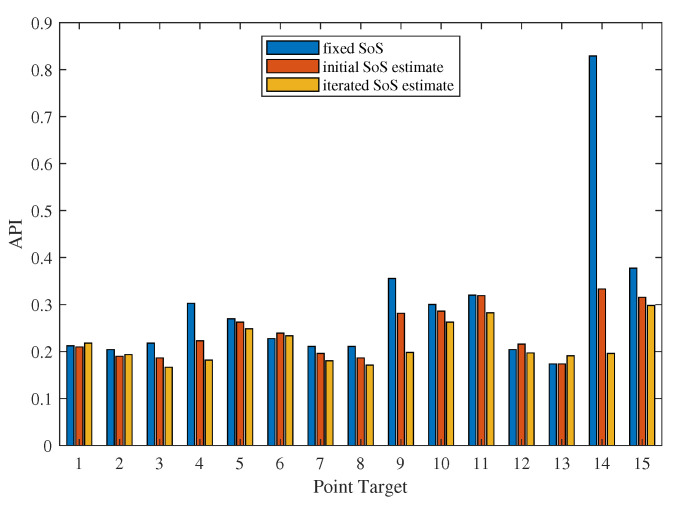
APIs of point target images beam-formed using different SoS estimates.

To quantitatively analyze how SoS estimates improve the quality of ultrasound imaging, the Array Performance Indexes (APIs) [32] of the 15 point targets were calculated. It can be seen that, for point targets located outside the defocused region, the image quality—as quantified by the API—remains relatively stable across different images (Figure 15). However, even with the initial SoS estimate, there is a notable improvement in the image quality for point targets within the defocused region. The API for the most severely defocused point target drops by 60% when initial estimate is used for imaging correction. Furthermore, employing iterated SoS estimates further enhances ultrasound image quality, with the APIs in these images showing a maximum decrease of 40% compared to those obtained with the initial estimate.

## 5. Conclusions

This paper introduces an iterative approach to ultrasound SoS estimation, enhancing ultrasound imaging correction. The proposed iterative pulse–echo ultrasound tomography algorithm (iterative CUTE) improves SoS estimation quality by iteratively correcting noise and errors that are inherent in single-pass approaches. Furthermore, this paper reveals that traditional precision quantification indicators, such as RMSE, do not effectively reflect the quality of SoS estimation for imaging correction. To quantitatively describe the quality of SoS estimation, the use coherence factor—as an indicator for evaluating improvements in imaging correction using SoS—was proposed. Performance validation of the iterative CUTE algorithm was conducted using simulations with phantoms containing variously shaped inclusions were and agar phantom experiments. The simulation results indicate that the iterative CUTE algorithm, in contrast to conventional single-pass SoS estimation algorithms, is significantly more proficient in rectifying errors present in the estimates produced by these existing methodologies. This leads to a marked enhancement in the quality of SoS estimations. Furthermore, in phantom experiments, the SoS estimate derived from the iterative CUTE algorithm demonstrated the ability to bring a clear improvement in the resultant images, especially when compared to those generated using existing algorithms. Such improvements not only underscore the algorithm’s efficacy in optimizing SoS estimations but also demonstrate its potential for significantly enhancing imaging corrections. The consistency of these findings with the results observed in simulation studies further corroborates the robustness and applicability of the iterative CUTE algorithm in both simulated and experimental scenarios. Although SoS estimate iteration convergence is achieved in both simulation and phantom experiments, a theoretical proof of such convergence is still required in ensuring the completeness of this algorithm. This will be the focus of our future work.

## Figures and Tables

**Table 1:** Different erroneous SoS estimate with the same RMSE of 9.7 m/s.

	Background SoS	Inclusion SoS
SoS estimate 1	1500 m/s	1540 m/s
SoS estimate 2	1407 m/s	1545 m/s
SoS estimate 3	1549.7 m/s	1549.7 m/s

## Data Availability

Data are contained within the article.
